# Tracking the epidemiological trends of female breast cancer in Saudi Arabia since 1990 and forecasting future statistics using global burden of disease data, time-series analysis

**DOI:** 10.1186/s12889-024-19377-x

**Published:** 2024-07-22

**Authors:** Ahmed Saad AL Zomia, Ibrahim Ali M AL Zehefa, Lama Ali Lahiq, Mohammed Tarek Mirdad, Abdullah Saad Alshahrani, Turki Alshahrani, Nawaf N. Almahfuth, Mahmoud Tarek Mirdad, Albara Awad Alqarni, Noor Mohamed Alshareef, Ryan M. AL Qahtani, Mohammed Abdulrahman Asiri, Mohammed Saad Alshahrani, Ramy Mohamed Ghazy, Ibrahim Tawhari

**Affiliations:** 1 College of Medicine University, Abha, Saudi Arabia; 2 Family and Community Medicine, College of Medicine, King Khalid University, Abha, Saudi Arabia; 3 Tropical Health Department, High Institute of Public Health, Alexandria University, Alexandria, Egypt; 4 Internal Medicine Department, College of Medicine, King Khalid University, Abha, Saudi Arabia

**Keywords:** Female breast cancer, Saudi Arabia, Time series, Forecasting, DALYs, Mortality

## Abstract

**Background:**

Female breast cancer stands as the prime type of cancer in the Kingdom of Saudi Arabia (KSA), with a high incidence and mortality rates. This study assessed the burden of female breast cancer in KSA by analyzing and forecasting its incidence, mortality, and disability-adjusted life years (DALYs).

**Methods:**

We retrieved data from the Global Burden of Disease (GBD) about female breast cancer from 1990 to 2021. Time-series analysis used the autoregressive integrated moving average (ARIMA) model to forecast female breast cancer statistics from 2022 to 2026.

**Results:**

From 1990 to 2021, KSA reported 77,513 cases of female breast cancer. The age groups with the highest number of cases are 45–49 years, followed by 40–44 years, 50–54 years, and 35–39 years. The analysis also showed fewer cases in the younger age groups, with the lowest number in the less than 20-year-old age group. From 1990 to 2021, KSA reported 19,440 deaths due to breast cancer, increasing from 201 cases in 1990 to 1,190 cases in 2021. The age-standardized incidence rate/100,000 of breast cancer increased from 15.4 (95% confidence interval (CI) 11.2–21.0) in 1990 to 46.0 (95%CI 34.5–61.5) in 2021. The forecasted incidence rate of female breast cancer will be 46.5 (95%CI 45.8–46.5) in 2022 and 49.6 (95%CI 46.8–52.3) in 2026. The age-standardized death rate per 100,000 Saudi women with breast cancer increased from 6.73 (95%CI 6.73–9.03) in 1990 to 9.77 (95%CI 7.63–13.00) in 2021. The forecasted female breast cancer death rate will slightly decrease to 9.67 (95%CI 9.49–9.84) in 2022 and to 9.26 (95%CI 8.37–10.15) in 2026. DALYs increased from 229.2 (95%CI 165.7–313.6) in 1990 to 346.1 (95%CI 253.9–467.2) in 2021. The forecasted DALYs of female breast cancer will slightly decrease to 343.3 (95%CI 337.2–349.5) in 2022 reaching 332.1 (95%CI 301.2–363.1) in 2026.

**Conclusions:**

Female breast cancer is still a significant public health burden that challenges the health system in KSA, current policies and interventions should be fashioned to alleviate the disease morbidity and mortality and mitigate its future burden.

**Supplementary Information:**

The online version contains supplementary material available at 10.1186/s12889-024-19377-x.

## Introduction

According to recent data, female breast cancer has surpassed lung cancer as the most diagnosed cancer worldwide. In 2020, there were an estimated 2.3 million new breast cancer cases, accounting for 11.7% of all newly diagnosed cancers. Unfortunately, breast cancer claimed the lives of 684,996 people in the same year [1]. There is a noteworthy discrepancy in breast cancer death rates between transitioning countries and transitioned countries, with transitioning countries having higher rates (15.0 per 100,000 vs. 12.8 per 100,000). [2]

Among the most frequently diagnosed cancers in the Kingdom of Saudi Arabia (KSA) are breast cancer, colorectal cancer, and prostate cancer. Breast cancer incidence is 14.8% (cumulative risk 2.87%) among both males and females. In 2018, the incidence of breast cancer among females was 29.7%. [3] In 2018, KSA recorded 10,518 cancer-related deaths and identified 24,485 new cancer cases in a population of 33,554,333. More than 50% of breast cancer cases in KSA are detected at a later stage, compared to 20% in developed countries. [4]

Various risk factors contribute to the development of female breast cancer, including reproductive and hormonal factors, smoking, lifestyle choices, and genetic predispositions. [5–8] About 8–10% of breast cancers are attributed to deleterious hereditary mutations, with BRCA1/2 mutations accounting for half of these. [9, 10] Different gene mutations are classified into high-, moderate- or low-penetrance groups, and risk reduction surgery may be considered based on the degree of penetration. [10, 11] Moreover, testing for germline BRCA1/2 mutations is crucial for identifying candidates who may benefit from specific treatments, such as poly (ADP-ribose) polymerase inhibitors or platinum drugs. [12] Abulkhair et al., [13] assessed the prevalence of BRCA1 and BRCA2 mutations in 310 Saudi patients, they found that 87% had no mutations. BRCA1 mutations were found in 11% of patients, while 2% had BRCA2 mutations. Triple-negative breast cancer (TNBC) was present in 86% of patients with mutations.

Despite the increased effectiveness of initial diagnostics and the rapid progress of pharmacotherapy in recent years, breast cancer remains the leading cause of mortality from malignant tumors in women worldwide. [14] The breast cancer mortality rate is 16.9 per 100 person-years observation, with a median survival time of 38.3 months. Predictors of survival include clinical stage III and IV, number of positive lymph nodes, co-morbidities, postmenopausal status, histologic grade III, and not receiving hormonal therapy. [15] Disability-adjusted life years (DALYs) are a time-based metric that measures the overall burden of disease in different populations. It combines years of life lost (YLLs) due to premature mortality and years lived with disability or in less than full health. This holistic approach helps policymakers and healthcare professionals prioritize interventions and allocate resources effectively. [16] In 2019, breast cancer accounted for 1,222,835 DALYs among female patients, with a range of 1,053,073 to 1,411,009. The majority of this burden (94.9%) was due to YLLs. [17] However, in most Western countries, the mortality rate for breast cancer decreased in recent years owing to advances in treatment strategies and early detection measures. [18, 19]

The promotion of preventive behaviors and the establishment of screening programs play a crucial role in reducing the incidence of breast cancer and facilitating early treatment. The Breast Health Global Initiative is currently responsible for preparing comprehensive guidelines and approaches to ensure effective breast cancer control globally. [20] Mammographic screening is regarded as the gold standard for breast cancer screening, lowering mortality by 23%. [21] Classical mammography has 75–95% sensitivity and 80–95% specificity. [22] Mammography was introduced in the KSA in 2002. Saudi’s Ministry of Health provides free mammography screenings. A nationwide breast cancer screening facility was established in 2007, and 1,215 people were screened for breast cancer in its first year of operation. [23]

This study was conducted to assess the burden of female breast cancer in KSA over 32 years (1990–2021) by analyzing the incidence, mortality, DALYs trend, and age-characteristic of female breast cancer among the Saudi population and forecast these metrics for the next five years.

## Methodology

To investigate the impact of female breast cancer in KSA, we utilized data from the Global Burden of Disease (GBD) spanning from 1990 to 2021. [24] The GBD study has been conducting annual estimations of various disease metrics since 1990, offering a valuable opportunity for comprehensive and comparable assessments of the burden and trends of diseases at global, regional, and national levels. We used data-driven metrics for breast cancer, including the age-standardized incidence rate per 100,000 population, breast cancer-related deaths, and DALYs. All these metrics were calculated as rates per 100,000 population to accurately represent the burden of the female breast cancer.

In this study, GBD data was subjected to thorough cleaning and preprocessing using R software version 4.2. To gain an initial understanding of the epidemiology of breast cancer in KSA, we performed a descriptive analysis of the number of cases reported since 1990 and the number of cases within each age group. We calculated the percentage change over the study period according to the following formula:

$$\eqalign{ Percentage\,change\, = \, & \cr & {\rm{(value}}\,{\rm{of}}\,{\rm{current\,year - value}}\,{\rm{of}}\,{\rm{the\,previous}}\,{\rm{year)/}} \cr & {\rm{(value}}\,{\rm{of}}\,{\rm{the}}\,{\rm{previous}}\,{\rm{year)}}\, \times {\rm{100\% }} \cr}$$

Age was clustered into 5 years, a total of 12 age groups were included starting from < 20 years and ending up with ≥ 70 years.

We used R packages, specifically “forecast” and “tseries,” to forecast the incidence and death rates for the next five years, from 2022 to 2026. This forecasting allows us to better comprehend potential trends and plan interventions accordingly. Line graphs were plotted with the X-Y axis, where X represented the years from 1990 to 2026, and Y represented the numerical or percentage values of the selected indicators.

A time series is a set of data points gathered, recorded, or observed at regular intervals. These data points are indexed in temporal order, usually at regular intervals (e.g., daily, monthly, yearly), and examined to discover patterns, trends, and dependencies throughout time. More specifically, they are used to forecast the values of a series using the autoregressive integrated moving average (ARIMA) model. [25] We used ARIMA to model and forecast female breast cancer statistics over the specified period. Autoregression order (AR(p)), moving average order (MA(q)), and the degree of difference (I(d)) were considered in our analysis. ARIMA models were constructed based on the breast cancer data reported from KSA from 1990 to 2021. These models were utilized to forecast age-standardized incidence, deaths, and DALYs (2022–2026). The key variables that entered the ARIMA model were:

- Year: The time variable representing each year from 1990 to 2021.
- Incidence rates: Annual breast cancer incidence rates, age-standardized per 100,000 population.
- Mortality rates: Annual breast cancer mortality rates, age-standardized per 100,000 population.
- Disability-Adjusted Life Years (DALYs): Annual DALYs associated with breast cancer.

The selection of the ideal model was guided by Akaike’s information criterion (AIC) and Schwartz’s Bayesian criterion (SBC) using autocorrelation function (ACF) and partial autocorrelation function (PACF) graphs to determine the degree of ARIMA. To ensure the reliability of our forecasts, we employed several validation measures. We checked that forecast errors (residuals) were uncorrelated using the Ljung-Box Q-test, assessed that the residuals followed a normal distribution with a mean of zero using the Shapiro-Wilk test, and verified that the residuals had constant variance through visual inspection of residual plots and the Breusch-Pagan test. [26]

## Results

### Female breast cancer incidence and deaths

**Fig. 1:**
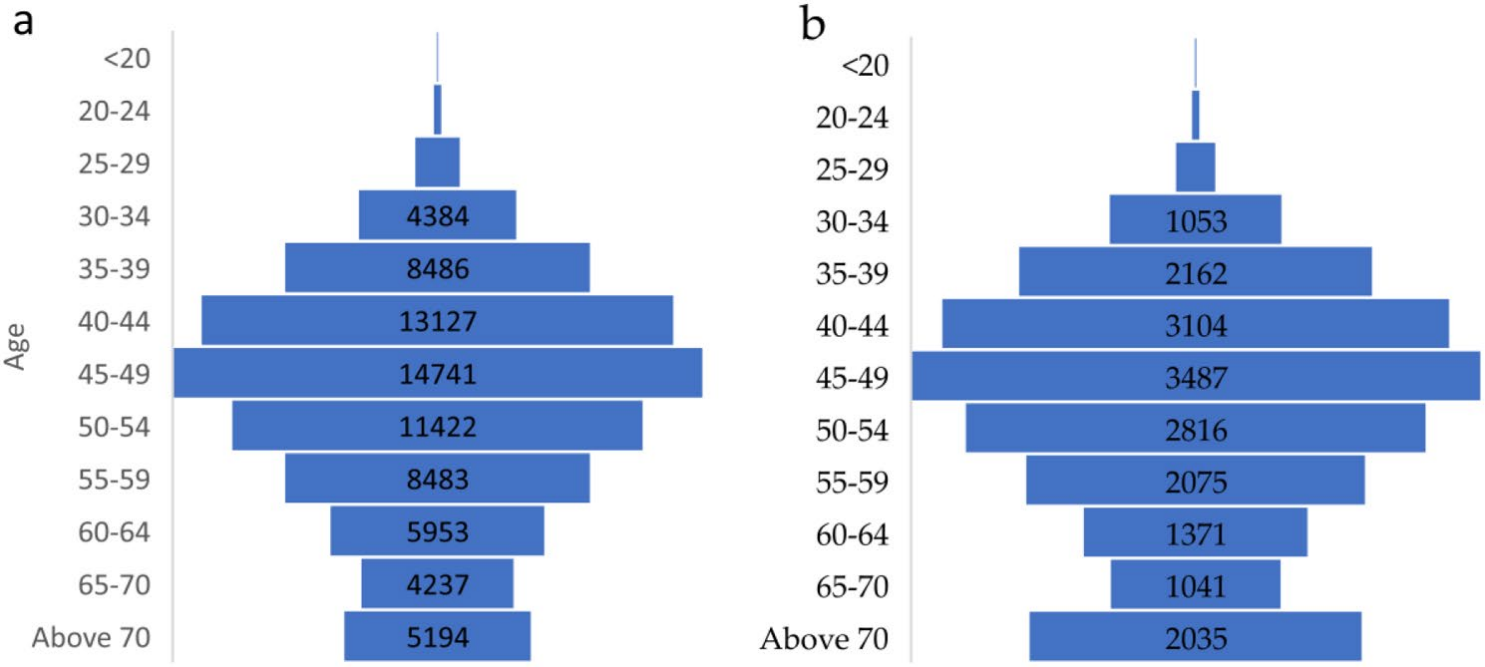
**a** Age distribution of female breast cancer incidence **b** mortality in Saudi Arabia due to female breast cancer(1990–2021)

From 1990 to 2021, KSA reported a total of 77,513 cases of female breast cancer. In 1990, there were 482 cases, and by 2021, this number had increased to 6,097 cases. The percentage change across different age groups is also presented in supplementary Table 1. Figure 1a illustrates the distribution of breast cancer cases across the various age groups since 1990. The data showed a discernible trend of increasing cases with advancing age. The age groups with the highest number of cases were 45–49 years (14,741 cases), followed by 40–44 years (13,127 cases), 50–54 years (11,442 cases), and 35–39 years (8,468 cases). The data also showed fewer cases in the younger age groups, with the lowest number in the age group of less than 20 years.

From 1990 to 2021, KSA reported a total of 19,440 deaths due to breast cancer. In 1990, there were 201 cases, which increased to 1,190 cases in 2021. The percentage change across different age groups is also presented in supplementary Table 2. Breast cancer mortality was highest in the 45–49 age group, with 3,487 deaths, followed by the 40–44 age group with 3,104 deaths. The 50–54 age group also showed high mortality, with over 2,800 deaths. Mortality rates decline in older age groups, though they remain substantial, with over 2,000 deaths in those aged above 70 years. Figure 1b.

**Fig. 2:**
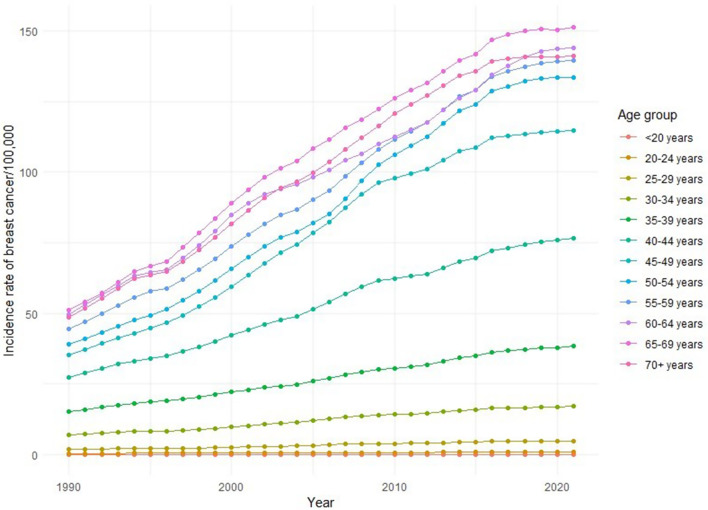
the incidence of female breast cancer across different age groups from 1990–2021

Figure 2 illustrates a significant increase in female breast cancer incidence rates across most age groups over the past three decades. Younger age groups, particularly those under 30, maintain low and stable incidence rates. Interestingly, the age group of 60 years and above shows the highest increase in incidence across the studied period.

**Fig. 3:**
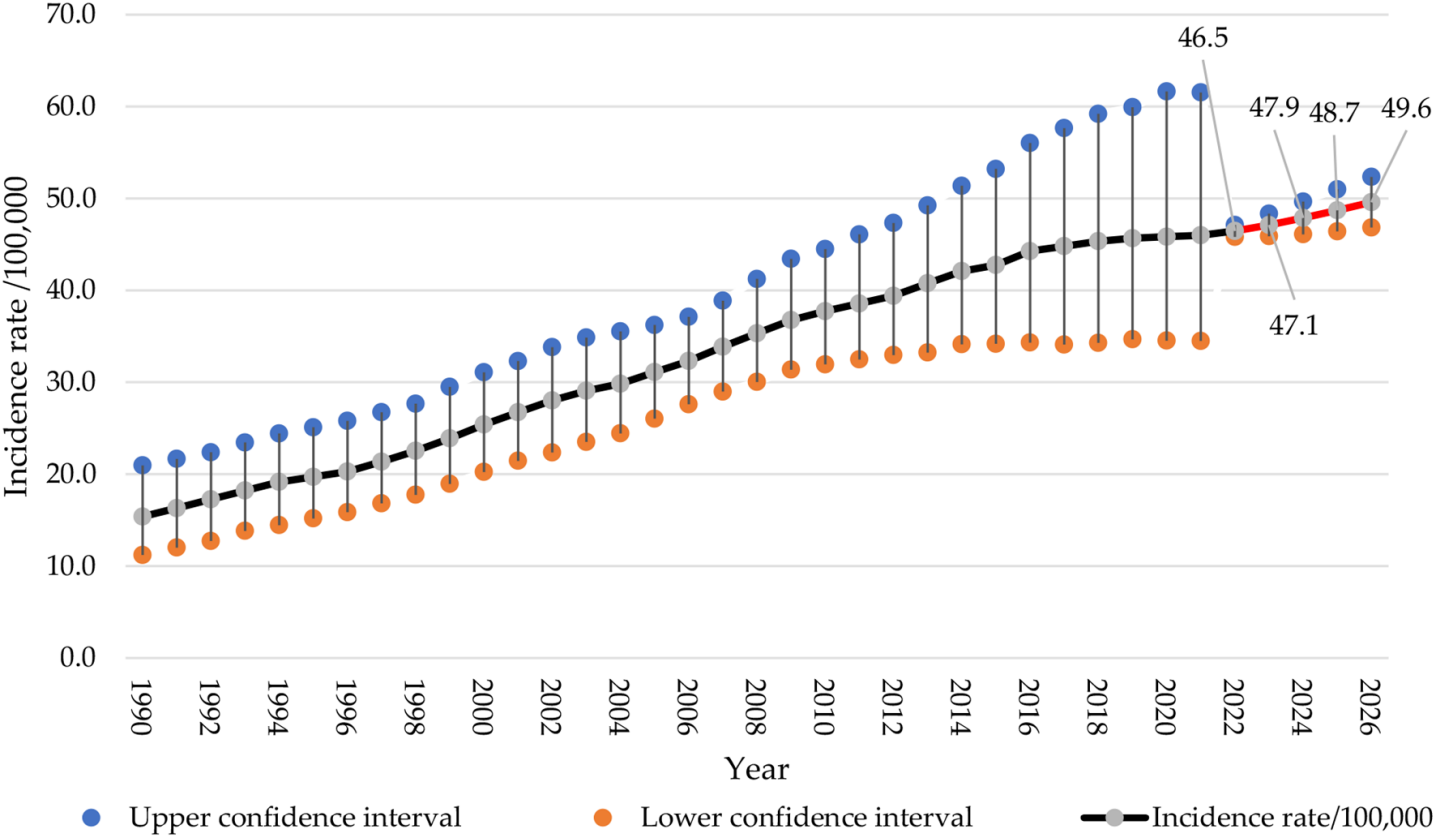
Female breast cancer age-standardized incidence rate in Saudi women was assessed from 1990 to 2021, alongside forecasted values for the period between 2022 and 2026 (red line)

The age-standardized incidence rate/100,000 of female breast cancer increased from 15.4 (95%CI 11.2–21.0) in 1990 to 46.0 (95%CI 34.5–61.5) in 2021. The forecasted incidence rate of breast cancer is 46.5 (95%CI 45.8–46.5) in 2022, followed by 47.1 (95%CI 45.8–46.5) cases in 2023. The forecast continues to show a slight increase, with an incidence rate of 47.9 (95%CI 46.1–49.7) cases forecasted in 2024, 48.7 (95%CI 46.4–51.0) for 2025, and 49.6 (95%CI 46.8–52.3) in 2026. Figure 3.

**Fig. 4:**
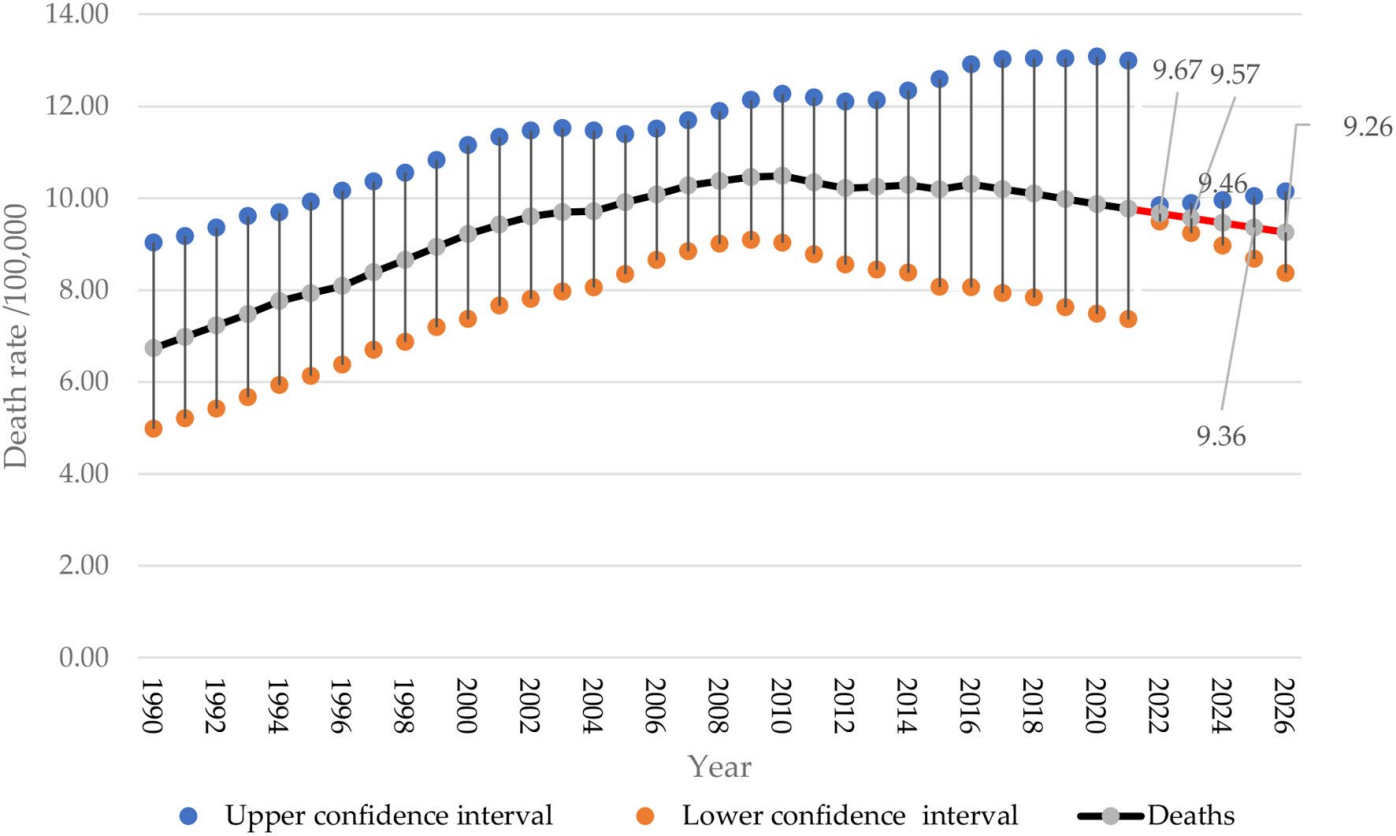
Breast cancer age-standardized deaths in Saudi women assessed from 1990 to 2021, alongside forecasted values for the period between 2022 and 2026 (red line)

The age-standardized death rate per 100,000 Saudi women with breast cancer is shown in Fig. 4. The death rate increased from 6.73 (95%CI 6.73–9.03) in 1990 to 9.77 (95%CI 7.63–13.00) in 2021. The forecasted death rate of breast cancer will slightly decrease to 9.67 (95%CI 9.49–9.84) in 2022, followed by 9.57 (95%CI 9.24–9.89) cases in 2023. The forecast continues to show a slight decrease to reach 9.46 (95%CI 8.97–9.96) in 2024, 9.36 (95%CI 8.68 − 10.04) in 2025, and 9.26 (95%CI 8.37–10.15) in 2026.

**Fig. 5:**
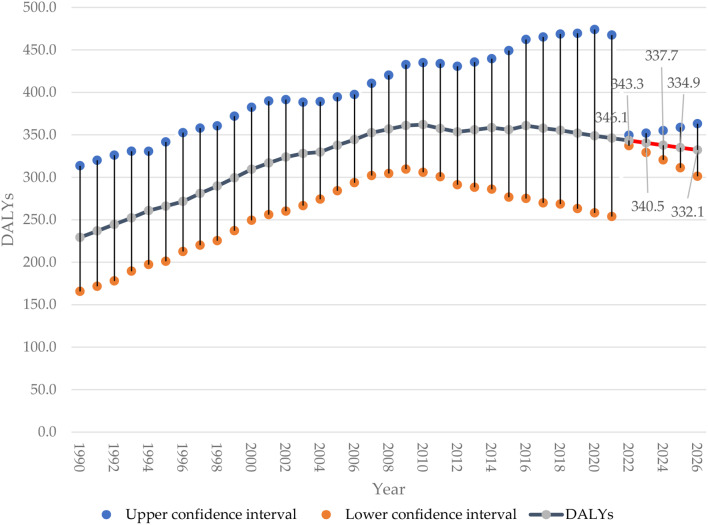
Breast cancer age-standardized DALYs in Saudi women assessed from 1990 to 2021, alongside forecasted DALYs values for the period between 2022 and 2026 (redline)

The DALYs for Saudi women with breast cancer are shown in Fig. 4. The DALYs increased from 229.2 (95%CI 165.7–313.6) in 1990 to 346.1 (95%CI 253.9–467.2) in 2021. In 2022, the forecasted DALYs of breast cancer will slightly decrease to 343.3 (95%CI 337.2–349.5), followed by 340.5 (95%CI 329.1–351.9) in 2023. The forecast continues to show a slight decrease to reach 337.7 (95%CI 320.5–355.0) DALYs in 2024, 334.9 (95%CI 311.1 − 358.7) in 2025, and 332.1 (95%CI 301.2–363.1) in 2026. Figure 5.

## Discussion

In this study, we utilized secondary data to detail the total number of reported female breast cancer cases and deaths within various age groups. Additionally, we described the age-standardized incidence, death, and DALYs. Finally, we made a forecast for the age-standardized incidence, death, and DALYs for the period from 2022 to 2026.

Indeed, the distinct patterns in female breast cancer incidence across different age groups underscore the significance of comprehending the disease’s trends within specific cohorts. This offers valuable information that can guide targeted approaches for screening, prevention, and treatment, ultimately leading to more effective strategies for addressing the burden of female breast cancer. In this study, breast cancer incidence was uncommon among females aged less than 20 years, but it exhibited a gradual increase with advancing age, revealing two distinct peaks in its distribution. The first and most prominent peak occurred among females aged 45–49 years. Additionally, a second, albeit less severe, peak was observed among females aged 40–44 years. This pattern is expected since breast cancer incidence generally rises with age, and it is consistent with what is reported in previous studies conducted in KSA [4, 27] and worldwide. [28, 29] On the other hand, previous studies in KSA have indicated that breast cancer tends to affect young females, with a median age of approximately 40 years at diagnosis [30, 31]. Breast cancer incidence shows a distinct age-specific curve, with a rapid increase before menopause, typically between ages 40 and 50, followed by a slower rate of increase. [32]. This slowdown is likely attributed to declining levels of circulating estrogens. [33] In countries with low breast cancer incidence, the curve’s slope after menopause may appear flat or even negative. This phenomenon results from increasing risks of occurrence in successive generations of women, rather than a true decline in risk with age. [28]

It is important to note that despite the highest number of cases being reported in the middle-aged population, the highest incidence per 100,000 breast cancer was found among those aged 60 years and above. This observation can be attributed, in part, to the population distribution, as a significant proportion of females fall within the middle-aged than older age groups. Likely, several epidemiological studies have reported that breast cancer tends to affect older women. For instance, a bimodal incidence pattern, similar to that observed in Western countries, is predicted in KSA shortly, with a second peak occurring in late life [34, 35]. This demographic factor plays a crucial role in understanding incidence patterns and helps to contextualize the age-specific distribution of breast cancer cases in the population.

The current analysis revealed a substantial and rapid increase in the number of reported female breast cancer cases and deaths each year in KSA, however, the annual percentage change in deaths and cases is decreasing across the study period. The age-standardized incidence of breast cancer reported per year escalated 4 times, rising from 15.4 in 1990 to 46.0 in 2021 and forecasted to be 49.6% in 2026. Likewise, the number of age-standardized deaths also showed a significant increase, growing by 1.5 times from 6.73 in 1990 to 9.77 in 2021 and forecasted to be 9.26 in 2026. Based on the forecast findings, the pattern of incidence is increasing, however, the death rate is decreasing. We speculate that this is due to Saudi ‘s continued efforts to implement public health programs focused on prevention, early detection, and effective treatment strategies to address the significant impact of female breast cancer. Additionally, improved access to healthcare services and the implementation of screening programs for early diagnosis and management of breast cancer in KSA have likely contributed to these trends. [36, 37]

There has been a growing emphasis on DALYs recognized as a common indicator to measure the burden of disease in many countries. [38] We found a significant increase in the DALYs, among females with breast cancer since 1990. Like the forecasted decline in deaths, DALYs are also expected to gradually decrease in the coming years. In the same line, Xu et al., [39] found that breast cancer-related deaths reached 700,660 globally, while the corresponding DALYs amounted to 20,625,313. Encouragingly, from 1990 to 2019, there was a decline in the age-standardized mortality rates and age-standardized DALYs’ rates for breast cancer on a global scale. [39] These findings underscore the importance of using DALYs as a comprehensive measure to understand the impact of breast cancer and to track its trends over time, contributing to evidence-based strategies and interventions for improved public health outcomes.

Implications of this study: The study underscores the need for targeted screening and early detection programs for women in KSA due to the rising number of breast cancer cases. Despite the forecasted reduction in mortality and DALYs, the projected increase in incidence through 2026 indicates a sustained burden. This necessitates ongoing efforts in prevention, education, and healthcare resource allocation to effectively address the continued impact of breast cancer.

### Strengths and limitations

This study used a large dataset that spanned a long period and included people of all ages. However, multiple limitations should be considered. The use of secondary data sources involves the possibility of shifts in data accuracy and completeness, which could affect the findings of the research. Specifically, this research is based on data extracted from the GBD data exchange repository, which has documented limitations in the sources and accuracy of recording and registration. Moreover, we were not able to determine the causal correlations between risk variables and breast cancer incidence or mortality . Furthermore, the study did not take into consideration particular risk factors for female breast cancer, such as genetics, lifestyle, and hormone use, all of which can have a major impact on disease incidence and outcomes. Additionally, while we used ARIMA models to forecast key female breast cancer metrics, these models did not include additional covariates outside of the time series data being forecasted. This focus on the temporal dynamics of breast cancer statistics means that potential external influencing factors were not incorporated into our forecast. Future studies should concentrate on identifying risk factors unique to the Saudi population to have a more complete picture of female breast cancer epidemiology in the region. Furthermore, primary data collection and longitudinal research could assist in validating these findings and better investigating causal links.

## Conclusions

By doing so, the research aimed to gain insights into the patterns and trends of female breast cancer statistics and identify potential areas that may require targeted interventions or improved screening and treatment measures. The analysis of female breast cancer cases over time showed a significant and consistent rise in the number of reported cases each year, indicating a growing burden of the disease. There is a clear age-specific pattern, with female breast cancer cases showing a notable increase with advancing age. The age groups with the highest number of cases were 45–49 years. Conversely, the younger age groups, particularly those under 20 years, exhibited the lowest number of cases. Forecasting for the years 2022 to 2026 suggests a continuation of this upward trend incidence despite the reduction in DALYs and mortality. These findings underscore the need for continued efforts to maintain breast cancer prevention, early detection and screening, and effective treatment strategies to address its significant impact on public health.

### Electronic supplementary material

Below is the link to the electronic supplementary material.

Supplementary Material 1

## Data Availability

Data was retrieved from the Global Burden of Disease Collaborative Network. Global Burden of Disease Study 2021 (GBD 2021) Results. Seattle, United States: Institute for Health Metrics and Evaluation (IHME), 2020. Available from https://vizhub.healthdata.org/gbd-results/.
