# Supplementary material for: Tracking the epidemiological trends of female breast cancer in Saudi Arabia since 1990 and forecasting future statistics using global burden of disease data, time-series analysis
Source: BMC Public Health. 2024 Jul 22;24:1953. doi: 10.1186/s12889-024-19377-x (PMC11265076; doi:10.1186/s12889-024-19377-x)
Supplement: Supplementary file 1 — Supplementary Material 1 [file 12889_2024_19377_MOESM1_ESM.docx]

**Supplementary Table 1: Number of deaths among females with breast cancer from 1990 to 2021**

| Age  Num | All age | <20 | 20-24 | 25-29 | 30-34 | 35-39 | 40-44 | 45-49 | 50-54 | 55-59 | 60-64 | 65-70 | above 70 | % change across all ages |
| --- | --- | --- | --- | --- | --- | --- | --- | --- | --- | --- | --- | --- | --- | --- |
|  | 201 | 0 | 1 | 3 | 12 | 22 | 30 | 29 | 23 | 19 | 17 | 12 | 33 |  |
|  | 217 | 0 | 1 | 3 | 13 | 24 | 32 | 31 | 25 | 21 | 18 | 13 | 35 | 8.0 |
|  | 235 | 0 | 1 | 4 | 14 | 26 | 34 | 34 | 27 | 23 | 19 | 15 | 37 | 8.0 |
|  | 253 | 0 | 1 | 4 | 15 | 28 | 37 | 37 | 30 | 26 | 20 | 17 | 39 | 7.8 |
|  | 274 | 0 | 1 | 4 | 16 | 30 | 39 | 40 | 33 | 28 | 22 | 20 | 41 | 8.1 |
|  | 291 | 0 | 1 | 4 | 16 | 32 | 42 | 43 | 35 | 31 | 23 | 22 | 43 | 6.5 |
|  | 311 | 0 | 1 | 4 | 17 | 34 | 46 | 46 | 38 | 32 | 24 | 23 | 44 | 6.6 |
|  | 335 | 0 | 1 | 4 | 18 | 36 | 49 | 50 | 42 | 35 | 27 | 25 | 48 | 7.8 |
|  | 359 | 0 | 1 | 5 | 19 | 37 | 52 | 54 | 45 | 38 | 30 | 26 | 52 | 7.3 |
|  | 386 | 0 | 1 | 5 | 20 | 40 | 56 | 58 | 49 | 41 | 33 | 28 | 56 | 7.5 |
|  | 415 | 0 | 1 | 5 | 21 | 42 | 59 | 64 | 53 | 45 | 36 | 28 | 60 | 7.4 |
|  | 441 | 0 | 1 | 5 | 22 | 45 | 63 | 70 | 56 | 47 | 37 | 30 | 64 | 6.3 |
|  | 467 | 0 | 1 | 6 | 23 | 47 | 66 | 77 | 60 | 50 | 39 | 32 | 66 | 6.0 |
|  | 491 | 0 | 1 | 6 | 25 | 49 | 70 | 82 | 64 | 52 | 40 | 33 | 68 | 5.0 |
|  | 511 | 0 | 1 | 6 | 26 | 51 | 74 | 87 | 67 | 54 | 41 | 34 | 69 | 4.2 |
|  | 543 | 0 | 2 | 7 | 29 | 55 | 81 | 93 | 72 | 57 | 42 | 35 | 71 | 6.3 |
|  | 576 | 0 | 2 | 8 | 32 | 60 | 87 | 100 | 78 | 59 | 43 | 35 | 72 | 5.9 |
|  | 613 | 0 | 2 | 8 | 35 | 65 | 94 | 109 | 85 | 63 | 44 | 35 | 73 | 6.5 |
|  | 646 | 0 | 2 | 9 | 37 | 70 | 101 | 117 | 92 | 66 | 44 | 35 | 74 | 5.4 |
|  | 681 | 0 | 2 | 9 | 39 | 75 | 107 | 126 | 98 | 70 | 45 | 35 | 74 | 5.5 |
|  | 714 | 0 | 2 | 10 | 41 | 80 | 112 | 134 | 104 | 75 | 46 | 36 | 74 | 4.8 |
|  | 738 | 0 | 2 | 10 | 42 | 84 | 117 | 139 | 109 | 79 | 47 | 36 | 74 | 3.3 |
|  | 764 | 0 | 2 | 10 | 43 | 88 | 122 | 144 | 114 | 83 | 48 | 36 | 73 | 3.6 |
|  | 809 | 0 | 2 | 11 | 46 | 94 | 131 | 152 | 122 | 88 | 51 | 37 | 74 | 5.8 |
|  | 856 | 0 | 2 | 11 | 49 | 100 | 141 | 161 | 132 | 92 | 54 | 38 | 75 | 5.9 |
|  | 895 | 0 | 2 | 11 | 50 | 105 | 150 | 168 | 139 | 96 | 57 | 40 | 75 | 4.6 |
|  | 956 | 0 | 2 | 12 | 53 | 113 | 163 | 180 | 150 | 103 | 62 | 42 | 76 | 6.8 |
|  | 998 | 0 | 2 | 12 | 54 | 117 | 172 | 190 | 158 | 108 | 66 | 44 | 77 | 4.4 |
|  | 1045 | 0 | 2 | 11 | 55 | 122 | 181 | 200 | 166 | 113 | 70 | 46 | 78 | 4.6 |
|  | 1090 | 0 | 2 | 11 | 56 | 126 | 189 | 211 | 174 | 120 | 73 | 48 | 79 | 4.4 |
|  | 1139 | 0 | 2 | 11 | 57 | 130 | 198 | 224 | 182 | 127 | 76 | 51 | 80 | 4.4 |
|  | 1190 | 0 | 2 | 11 | 57 | 135 | 207 | 237 | 192 | 134 | 80 | 54 | 81 | 4.5 |

**Supplementary Table 2: Number of cases among females with breast cancer from 1990 to 2021**

| Age  Num | all ages | <20 | 20-24 | 25-29 | 30-34 | 35-39 | 40-44 | 45-49 | 50-54 | 55-59 | 60-64 | 65-70 | Above 70 | % change across all ages |
| --- | --- | --- | --- | --- | --- | --- | --- | --- | --- | --- | --- | --- | --- | --- |
|  | 482 | 0 | 2 | 10 | 31 | 54 | 77 | 73 | 58 | 47 | 44 | 31 | 56 |  |
|  | 534 | 1 | 2 | 10 | 34 | 61 | 84 | 82 | 64 | 53 | 48 | 35 | 60 | 10.7 |
|  | 591 | 1 | 2 | 11 | 37 | 68 | 93 | 91 | 71 | 61 | 53 | 40 | 64 | 10.8 |
|  | 652 | 1 | 3 | 12 | 40 | 74 | 101 | 100 | 78 | 68 | 58 | 47 | 69 | 10.3 |
|  | 716 | 1 | 3 | 13 | 44 | 80 | 110 | 110 | 87 | 77 | 63 | 55 | 74 | 9.8 |
|  | 770 | 1 | 3 | 14 | 46 | 85 | 118 | 120 | 95 | 84 | 65 | 61 | 78 | 7.5 |
|  | 829 | 1 | 3 | 14 | 49 | 92 | 130 | 130 | 105 | 89 | 70 | 65 | 82 | 7.7 |
|  | 909 | 1 | 3 | 15 | 52 | 98 | 142 | 143 | 116 | 98 | 81 | 71 | 91 | 9.6 |
|  | 998 | 1 | 3 | 16 | 55 | 105 | 155 | 158 | 127 | 108 | 91 | 77 | 102 | 9.8 |
|  | 1102 | 1 | 4 | 18 | 60 | 114 | 169 | 175 | 142 | 121 | 103 | 83 | 114 | 10.4 |
|  | 1219 | 1 | 4 | 19 | 65 | 125 | 185 | 198 | 158 | 134 | 114 | 87 | 127 | 10.6 |
|  | 1334 | 1 | 4 | 21 | 71 | 135 | 200 | 224 | 173 | 147 | 124 | 96 | 139 | 9.5 |
|  | 1455 | 1 | 5 | 23 | 77 | 145 | 218 | 251 | 190 | 159 | 132 | 105 | 148 | 9.1 |
|  | 1570 | 1 | 5 | 25 | 84 | 156 | 237 | 277 | 207 | 170 | 140 | 112 | 156 | 7.9 |
|  | 1676 | 1 | 5 | 28 | 91 | 167 | 256 | 300 | 222 | 180 | 147 | 117 | 161 | 6.8 |
|  | 1821 | 1 | 6 | 31 | 103 | 183 | 285 | 329 | 243 | 195 | 155 | 123 | 166 | 8.6 |
|  | 1973 | 1 | 7 | 35 | 116 | 202 | 314 | 361 | 269 | 207 | 161 | 126 | 172 | 8.4 |
|  | 2161 | 2 | 7 | 39 | 129 | 225 | 350 | 403 | 301 | 227 | 169 | 130 | 179 | 9.5 |
|  | 2357 | 2 | 8 | 42 | 141 | 249 | 386 | 449 | 337 | 248 | 176 | 135 | 185 | 9.1 |
|  | 2569 | 2 | 8 | 45 | 154 | 275 | 423 | 498 | 373 | 272 | 187 | 142 | 190 | 9.0 |
|  | 2758 | 2 | 9 | 49 | 165 | 301 | 455 | 538 | 401 | 297 | 199 | 148 | 193 | 7.4 |
|  | 2960 | 2 | 9 | 52 | 178 | 329 | 491 | 579 | 435 | 324 | 209 | 154 | 197 | 7.3 |
|  | 3178 | 2 | 9 | 56 | 190 | 356 | 531 | 623 | 473 | 352 | 223 | 160 | 203 | 7.4 |
|  | 3474 | 2 | 10 | 61 | 208 | 394 | 588 | 681 | 522 | 384 | 244 | 170 | 211 | 9.3 |
|  | 3788 | 2 | 10 | 65 | 225 | 432 | 651 | 741 | 577 | 417 | 266 | 182 | 220 | 9.0 |
|  | 4066 | 2 | 10 | 68 | 239 | 465 | 710 | 793 | 627 | 444 | 290 | 194 | 224 | 7.3 |
|  | 4450 | 2 | 11 | 72 | 257 | 507 | 788 | 870 | 692 | 486 | 324 | 208 | 234 | 9.4 |
|  | 4760 | 2 | 11 | 73 | 270 | 539 | 851 | 935 | 744 | 522 | 351 | 222 | 241 | 7.0 |
|  | 5091 | 2 | 11 | 74 | 281 | 572 | 916 | 1008 | 800 | 562 | 379 | 238 | 250 | 7.0 |
|  | 5421 | 1 | 11 | 75 | 290 | 602 | 977 | 1084 | 855 | 606 | 405 | 254 | 260 | 6.5 |
|  | 5752 | 1 | 10 | 75 | 299 | 633 | 1037 | 1167 | 909 | 650 | 428 | 273 | 270 | 6.1 |
|  | 6097 | 1 | 10 | 76 | 305 | 664 | 1097 | 1251 | 970 | 695 | 454 | 295 | 279 | 6.0 |
